# Association of the presence of a COVID-19 infection at the time of birth and the rates of exclusive breastfeeding upon discharge in BFHI hospitals: a multicenter, prospective cohort study

**DOI:** 10.1186/s13006-023-00590-0

**Published:** 2023-10-04

**Authors:** Miguel Ángel Marín Gabriel, Sergio Martín Lozoya, Susana de las Heras Ibarra, Laura Domingo Comeche, Ersilia González Carrasco, Paula Lalaguna Mallada, Natalia Villó Sirerol, Lucía García Fernández, José Jiménez Martínez, Ana Royuela Vicente

**Affiliations:** 1 Department of Pediatrics, Puerta de Hierro-Majadahonda University Hospital, BFHI Hospital-Coordinator, Majadahonda, Madrid, Spain; 2 Department of Neonatology, Fuenlabrada University Hospital, Fuenlabrada, Madrid, Spain; 3 Department of Neonatology, Severo Ochoa University Hospital, Leganés, Spain; 4 Department of Pediatrics, Barbastro Hospital, Huesca, Spain; 5 Department of Pediatrics, Sanitas-La Zarzuela Hospital, Madrid, Spain; 6 Department of Pediatrics, Sanitas-La Moraleja Hospital, Madrid, Spain; 7 Biostatistics Unit, Hospital Universitario Puerta de Hierro Majadahonda, IDIPHISA. CIBERESP, ISCIII., Madrid, España

**Keywords:** Breastfeeding, COVID-19, Baby Friendly Hospital Initiative, Cesarean

## Abstract

**Background:**

Very few studies have assessed the association between COVID-19 infection and the rates of exclusive breastfeeding (EBF) upon discharge following the first waves of the pandemic and after initiation of vaccination. The primary objective of this study is to compare the rates of EBF since birth upon discharge in mothers diagnosed with COVID-19 infection at the time of the delivery versus a group of non-infected mothers in maternity hospitals with Baby Friendly Hospital Initiative (BFHI) accreditation. The secondary objectives include determining the rates of any breastfeeding at three and six months of life in both groups, as well as determining the possible factors associated with EBF rates observed upon discharge.

**Methods:**

An observational, Spanish multi-center hospital, prospective cohort study conducted from 1 to 2021 to 31 March 2022 and with follow-up during the first six months of life. Follow-up was performed via telephone contact with calls performed at three and six months. A multivariate logistic regression analysis model was used to identify the factors related to a lower probability of EBF upon discharge.

**Results:**

308 mother-infant pairs participated in the study, 111 in the cohort of women with COVID infection and 197 in the comparison group. EBF upon discharge was 62.7% in the COVID group vs. 81.2% in the comparison group (*p* = 0.002); at three months; 52.4% vs. 57.0% (*p* = 0.33) were performing EBF, with the rates of EBF at six months being 43.0% vs. 39.3% (*p* = 0.45), respectively. Exposure to COVID-19 at delivery (AOR 5.28; 95% CI 2.01, 13.86), not practicing BF previously (AOR 36.3; 95% CI 7.02, 187.74), birth via Cesarean section (AOR 5.06; 95% CI 1.62, 15.79) and low birth weight of the newborn (AOR 1.01; 95% CI 1.01, 1.01) were associated with a greater risk of not performing EBF upon discharge.

**Conclusions:**

Mothers with a mild or asymptomatic COVID-19 infection at the time of the delivery were less likely to have exclusively breastfed during their hospital stay than other mothers in these BFHI-accredited hospitals. However, there were no differences in breastfeeding rates between the groups at three and six months postpartum.

## Background

One of the strategies that has been supported by evidence to be effective in the initiation and maintenance of breastfeeding is the implementation of the Baby Friendly Hospital Initiative (BFHI) via the ten steps to breastfeeding success which include, among others, the facilitation of immediate and uninterrupted skin-to-skin contact following birth, rooming-in 24 h a day, and supporting mothers to recognize and respond to their infants’ cues for feeding. There is scientific evidence that shows an improvement in the rates of breastfeeding as progress is made to comply with the various steps suggested by the BFHI [1–3]. The IHAN (Initiative for Humanizing Birth and Breastfeeding Care) is a Spanish non-profit organization that is in charge of implementing the best quality standards in perinatal practice, including the protection and support of breastfeeding in maternity wards and primary healthcare centers, and is also responsible for the implementation of the BFHI. In Spain, the BFHI accreditation for maternity wards is carried out in four phases: In phase 1D (Discovery), hospitals commit to adjusting their practices and to setting up a breastfeeding committee. In phase 2D (Development), the various documents and tools needed to initiate the proposed changes are created. In phase 3D (Dissemination), the hospitals implement the plan created in the previous phase. They disseminate the breastfeeding policy, train and perform skills assessments of their professionals, draw up various protocols for perinatal care, and monitor practices by providing mothers and pregnant patients with surveys. Finally, in phase 4D (Designation), an external assessment is conducted to prove compliance with the quality requirements, which include, among others, the presence of exclusive breastfeeding rates upon discharge of at least 75% [4].

The COVID-19 pandemic has been a major challenge for clinical management in various areas, with perinatal care being one of the most affected areas due to the initial lack of knowledge regarding how this disease was transmitted, as well as the possible impact it could have on an especially vulnerable population, such as newborns. Initially, the Centers for Disease Control and Prevention (CDC) and the American Academy of Pediatrics (AAP) published guides on the management of infants born to mothers with a confirmed or suspected COVID-19 infection, which included the temporary separation of the mother-infant pairs in its guidelines. With regard to breastfeeding, given the initial lack of knowledge on the possibility of infecting the newborn via this route, the first recommendations included administering artificial formula or breast milk extracted from authorized caregivers [5, 6]. In contrast to these recommendations, the World Health Organization (WHO), United Nations Children’s Fund (UNICEF), and in Spain, the Ministry of Health, published various documents which supported maintaining early mother-baby contact and avoiding separation and which were in favor of providing support for breastfeeding mothers with the infection while ensuring the use of hygienic measures and face masks [7–9]. As time went on, scientific evidence showed that the risk of vertical transmission was very low, with neonatal infections usually being asymptomatic or presenting few symptoms [10, 11]. With regard to breastfeeding, various studies have noted the absence of viable virus in samples of breast milk as well as its transmission by this route [12, 13].

During the first waves of the pandemic and due, on many occasions, to the pressure on healthcare and the reorganization of hospital activities, it became more difficult to comply with perinatal care protocols, even in BFHI-accredited hospital centers [14, 15]. This resulted in a reduction in the rates of exclusive breastfeeding upon discharge with figures below the requirements established by the BFHI compared to historical cohorts (prior to COVID-19) [14, 16–18]. To date, there have been very few prospective studies conducted that compare the rates of exclusive breastfeeding in mother-infant pairs whose mothers presented with a positive diagnosis for a COVID-19 infection at the time of the birth versus those who did not present with such a diagnosis following the first waves of the pandemic and after changes began at a preventative level with the initiation of vaccination.

Given the need to isolate, which restricted the movement of these mothers once they had been diagnosed with the infection, it is also likely that they received less support following their discharge from hospital, and in such cases they were only able to access off-site resources (for example, breastfeeding support groups or medical follow-up via telephone or video) [19–21].

## Methods

### Study sample and setting

The primary objective of this research is to compare the rates of exclusive breastfeeding since birth upon discharge in mothers diagnosed with a COVID-19 infection at the time of the delivery versus a group of non-infected mothers in maternity hospitals with full BFHI accreditation (phase 4D). The secondary objectives include determining the rates of any breastfeeding at three and six months of life in both groups, as well as determining the possible factors that are associated with the rates of exclusive breastfeeding observed upon discharge.

An observational, Spanish multi-center hospital, prospective cohort study was conducted over a period of one year (from 1 to 2021 to 31 March 2022) and with follow-up during the first six months of life. Mothers who had been diagnosed with a COVID-19 infection at the time of the birth via a PCR screening and / or antigen determination and with a mild or asymptomatic reaction were included in the group of COVID mothers. Convenience sampling was performed for the group of non-COVID mothers, for which those selected were the mothers who had not been diagnosed with a COVID-19 infection at delivery and who delivered immediately before or after the positive COVID-19 mother.

A physician from each of the hospitals included in the study was in charge of collecting the data as well as the telephone follow up. Patient confidentiality was maintained by a numerical code assigned by the study coordinator.

The protocol was approved by the Ethics Committees of all participating hospitals and was performed in accordance with the ethical standards of the Declaration of Helsinki.

During the study period, the fourth, fifth and sixth waves of the COVID-19 pandemic took place. During this time, the recommendations of the Ministry of Health were upheld in relation to birth, consisting, among others, in the application of immediate skin-to-skin contact, rooming-in of the mother-newborn pair and the administration of breastfeeding directly from the breast using suitable protection measures (hand hygiene and the use of masks).

With regard to the prevention program through the administration of the vaccine in Spain, during this period, healthcare staff and health and social care staff had already been administered the first two vaccine doses.

### Measures

Immediate skin-to-skin implementation after birth was defined as early and uninterrupted skin-to-skin contact with the mother after birth, and offering help for the initiation of breastfeeding as soon as possible. Rooming-in was considered to have been implemented when mothers and their infants were allowed to remain together 24-hours a day. Companionship was defined as the uninterrupted presence of a companion throughout the birth process.

Exclusive breastfeeding was defined as feeding the infant with human milk without supplementation during the entire hospital stay (e.g., infant formula or other human milk replacements); breastfeeding was defined as feeding with human milk combined with infant formula; and formula feeding when infants were fed exclusively with infant formula.

The variables related to mothers included in the study were the age of the mother (years), the level of studies (no education, primary, secondary or university education), marital status (single or not), breast reduction surgery (yes or no), previous children (yes or no) and the type of feeding (any breastfeeding or formula feeding), satisfactory (yes or no), and duration of feeding these children were given, information on breastfeeding received on a prenatal basis and during the stay in the delivery room (yes or no), type of feeding desired during pregnancy (exclusive breastfeeding, breastfeeding, formula feeding), type of birth (vaginal or Cesarean delivery), the presence of companionship during dilation and birth (yes or no), the administration of epidural analgesics (yes or no), and the observation of at least one attempt of breastfeeding during the mother’s hospital stay (yes or no). All mothers were asked if they thought that a woman with a COVID-19 infection could breastfeed while using protection measures. With regard to the newborn, the variables were sex, gestational age in weeks, birth weight in grams, the application of immediate skin-to-skin contact (yes or no), the need for advanced resuscitation (administration of positive or higher pressure) and the conduct of rooming-in (yes or no).

Follow-up was performed via telephone contact with calls being conducted at three and six months. Participants were considered to be lost to follow-up when three unsuccessful attempts to contact them had been made. In the contact conducted at three months of life, participants were asked about the type of feeding administered upon hospital discharge (exclusive breastfeeding, breastfeeding, formula feeding), the use of a pacifier (yes or no), the need for hospital follow-up or follow-up in a primary care facility following discharge from the maternity ward (yes or no), and contact with support groups (yes or no). In all cases, participants were asked whether the follow-up was performed face-to-face or virtually (by telephone and / or video). Participants were also asked about the type of feeding at the time (exclusive breastfeeding, breastfeeding, formula feeding), return to work (yes or no), conditions aimed at facilitating the continuation of breastfeeding (flexible schedule or not) and how it was carried out (on-site, remote working, or hybrid). At the six-month follow-up, participants were asked about the type of feeding provided at the time (exclusive breastfeeding, breastfeeding, formula feeding), their return to work and how it was carried out, as well as about the baby attending a daycare center (yes or no).

### Study protocol

Seven maternity wards with BFHI accreditation were invited to participate in the study, although one of them was unable to participate due to structural reasons. With regard to the cohort of COVID patients, 111 mothers participated, versus 197 in the comparison group. No mother-infant pair rejected being enrolled in the study. Eligible mothers were informed about the study and gave their informed consent while they were in the maternity ward. Informed consent could not be obtained for 25 women in the comparison group, already having been discharged at the time of the recruitment proposal.

Maternal exclusion criteria in both groups were the presence of a language barrier, the impossibility of guaranteeing post-discharge follow-up, the admission of the mother to the ICU, or if informed consent was not granted. Exclusion criteria for the newborns in both groups were prematurity, the birth of twins, and the admission of the newborn to the neonatal ward following birth due to a health issue.

### Data analysis

The quantitative variables are expressed as mean and median, and measures of dispersion (standard deviation and interquartile range). The qualitative variables are expressed as absolute frequencies and percentages. The assumption of normality was evaluated using the Shapiro-Wilk test. Differences between both cohorts were evaluated using the Student’s t-test or Mann–Whitney U test in order to contrast numerical variables. The Chi-squared test or Fisher’s exact statistical test were used for the categorical variables.

A univariate analysis was performed via binary logistic regression in order to understand the factors associated with not performing exclusive breastfeeding upon discharge. A multivariate logistic regression model was drawn up with the variables that were found to be significant in the univariate analysis and with those that are clinically relevant based on the literature. A backward stepwise regression strategy was conducted so that those variables with a value of *p* < 0.05 remained in the final model. The odds ratios (ORs) and their corresponding 95% confidence intervals (95% CI) were presented as effect measures. Values of *p* < 0.05 were deemed to be statistically significant. The software used for the analysis was Stata v.17.

## Results

Three-hundred and eight mother-infant pairs initially participated in the study, 111 in the cohort of women with COVID infection and 197 in the comparison group. Of these, nine were excluded due to the newborn being admitted for clinical reasons in the first hours of life: one in the COVID group (0.9%) and eight in the comparison group (4.0%). Table 1 lists the demographic data and variables related to the perinatal care of both groups.

**Table 1:** Demographic characteristics of the sample under study

	COVID patients (*n* = 110)	Comparison group (*n* = 189)	*P*-value
***Maternal characteristics***, *(%)*			
Age in years, median (IQR)	33 (29–36)	35 (31–37)	0.005
Maternal education			0.001
No education	0.9	1.1	
Primary education	16.7	5.0	
Secondary education	44.2	37.7	
University education	38.2	56.2	
Marital status (single)	4.8	4.4	0.87
Breast reduction surgery	1.9	2.7	0.65
Previous children	60.9	55.5	0.36
Previous any BF	88.8	92.1	0.47
Satisfactory previous BF	80.0	83.8	0.55
Duration of previous BF in weeks, median (IQR)	28 (16–79)	32 (20–68)	0.66
Type of feeding desired during pregnancy			0.04
EBF	84.1	92.9	
BF	6.6	2.2	
FF	9.3	4.9	
Vaginal birth	74.3	80.4	0.21
Epidural analgesia	89.6	91.9	0.50
***Variables related to the newborn***			
Sex (female)	60.0	50.2	0.10
Gestational age in weeks, median (IQR)	39 (38–40)	39 (38–40)	0.93
Weight in grams, median (IQR)	3230 (3010–3500)	3230 (3010–3560)	0.78
Advanced resuscitation	9.2	5.3	0.19
***Variables related to perinatal care***			
Information on BF in prenatal consultations	71.8	73.7	0.67
Information on BF in the delivery room	89.9	86.2	0.43
Companionship during dilatation	92.4	95.6	0.25
Companionship during the birth	77.1	86.2	0.04
Skin-to-skin contact in the delivery room	89.8	93.1	0.31
Rooming-in	100	99.4	0.44
Assessment of at least one intake during the hospital stay	87.9	93.5	0.25

*BF* breastfeeding; *EBF* exclusive breastfeeding; *FF* formula feeding; *IQR* interquartile range

When the mothers were asked if they thought a mother with a COVID-19 infection could directly breastfeed a newborn using protective measures, 84.7% of mothers with a COVID-19 infection and 83.0% of the mothers without an infection at delivery believed it was a safe way of providing breastfeeding (*p = 0.92*).

With regard to the follow-up conducted at three months, 9 (8.1%) mother-infant pairs were lost to follow-up in the COVID group and 18 (9.5%) in the comparison group (*p = 0.69*). At six months, 18 (16.3%) mother-infant pairs were lost to follow-up in the COVID group and 26 (13.7%) in the comparison group (*p = 0.53*).

Table 2 lists the data corresponding to the calls conducted at three and six months. At three months of age, 13 mothers who formed part of the COVID group had gone back to work, with seven of them (53.8%) reporting conditions aimed at facilitating the continuation of breastfeeding, versus seven mothers who formed part of the comparison group, with five of them (71.4%) reporting conditions aimed at facilitating the continuation of breastfeeding (*p = 0.007*). At six months of age, 43 mothers from the COVID group had gone back to work, with 22 of them (51.1%) reporting conditions aimed at facilitating the continuation of breastfeeding, versus 87 mothers from the comparison group, with 45 of them (51.7%) reporting conditions aimed at facilitating the continuation of breastfeeding *(p = 0.28*).

**Table 2:** Demographic characteristics at three- and six-month follow-up. Values given as percentage and median (interquartile range)

	COVID patients	Comparison group	*P*-value
***Follow-up at 3 months***	*n* = 101	*n* = 171	
Use of pacifier	68.0	72.0	0.52
Hospital follow-up after discharge			< 0.0001
On-site	22.0	17.8	
Remote	23.0	0.0	
Not specified	55.0	82.2	
Follow-up in a primary care facility after discharge			0.008
On-site	70.7	86.3	
Remote	4.0	1.8	
Not specified	25.3	11.9	
Attendance of a support group			0.42
On-site	14.0	14.9	
Remote	3.0	6.6	
Did not attend	83.0	78.5	
Going back to work	12.8	4.1	0.008
Form of professional activity			0.03
On-site	84.6	71.4	
Remote	15.4	14.3	
Hybrid	0.0	14.3	
Type of feeding upon discharge			0.002
EBF	62.7	81.2	
BF	24.5	10.3	
FF	12.8	8.5	
Type of feeding at 3 months			0.33
EBF	52.4	57.0	
BF	17.8	21.2	
FF	29.8	21.8	
***Follow-up at 6 months***	*n* = 92	*n* = 163	
Going back to work	46.7	53.3	0.30
Form of professional activity			0.21
On-site	81.4	70.1	
Remote	11.6	10.3	
Hybrid	7.0	19.6	
First attendance at a daycare center	12.0	9.9	0.59
Age (w) upon first attendance at a daycare center	20 (16–23)	20 (16–23)	1
Type of feeding at 6 months			0.45
EBF	43.0	39.3	
BF	16.1	22.7	
FF	40.9	38.0	

*BF* breastfeeding; *EBF* exclusive breastfeeding; *FF* formula feeding;

*w* weeks

A univariate analysis (Table 3) was performed in order to identify the factors associated with not exclusively breastfeeding upon discharge in both groups.

**Table 3:** Factors associated with non-exclusive breastfeeding at discharge: results of univariate analysis

	OR (95% CI)	*P*-value
*Variables related to the mother*		
COVID-19 at delivery	2.63 (1.49, 4.44)	0.001
Maternal age	1.03 (0.98, 1.08)	0.23
No university education	1.26 (0.72, 2.19)	0.41
Marital status (single)	0.9 (0.24, 3.4)	0.88
No previous children	1.05 (0.61, 1.78)	0.87
No previous practice of BF	12.1 (3.08, 47.5)	< 0.0001
Desire during pregnancy to practice BF or FF	12.6 (2.48, 64.7)	0.002
Cesarean	3.78 (2.08, 6.85)	< 0.0001
Epidural analgesia	1.96 (0.65,5.91)	0.23
***Variables related to the newborn***		
Sex (female)	1.36 (0.79, 2.32)	0.25
Gestational age	0.78 (0.63, 0.98)	0.03
Low birth weight (g)	1.01 (1.01, 1.01)	0.008
***Variables related to perinatal care***		
No information on BF in prenatal consultations	0.91 (0.45, 1.85)	0.81
No information on BF in the delivery room	1.08 (0.47, 2.44)	0.19
No companionship during the birth	2.26 (1.17, 4.36)	0.01
No immediate skin-to-skin contact performed	2.26 (0.99, 5.19)	0.05
No observation of at least one BF attempt during the hospital stay	1.5 (0.13, 13.27)	0.79

*BF* breastfeeding; *FF* formula feeding;

*g* grams

The variables which were entered into the multivariable logistic model were: exposure to COVID-19 at delivery; not having previously practiced breastfeeding; the desire during pregnancy to not provide exclusive breastfeeding; birth via Cesarean section; gestational age and low birth weight; not having companionship during the birth; and not applying immediate skin-to-skin contact. The variable concerning the desire during pregnancy to not provide exclusive breastfeeding was discarded due to collinearity with the type of feeding variable. After the backward stepwise selection, the following variables remained in the model: exposure to COVID-19 at delivery (AOR 5.28); not having previously practiced breastfeeding (AOR 36.3), birth via Cesarean section (AOR 5.06); and the low birth weight of the newborn (AOR 1.01) (Table 4).

**Table 4:** Factors associated with non-exclusive breastfeeding at discharge: results of multivariable logistic regression

	AOR (95% CI)	*P*-value
COVID-19 at delivery	5.28 (2.01, 13.86)	0.001
No previous practice of BF	36.3 (7.02, 187.74)	< 0.0001
Cesarean	5.06 (1.62, 15.79)	0.005
Low birth weight (g)	1.01 (1.01, 1.01)	0.006

*EBF* exclusive breastfeeding; *g* grams

## Discussion

In this study conducted in BFHI-accredited maternity wards, it was observed how a mild or asymptomatic COVID-19 infection at the time of the delivery was associated with a lower probability of exclusive breastfeeding at the time of discharge from hospital. In the study conducted by Zanardo *et a*l, it was observed how during the first months of the pandemic in Italy (during lockdown) the rates of exclusive breastfeeding upon discharge in a hospital center were 70.3% Vs. 86.3% in a historic control group from the previous year [16]. However, the maternal COVID-19 infection status was not assessed by this study group; a limitation that we also found in the study conducted by Preszler et al. in which they obtained exclusive breastfeeding rates of 78.2% Vs 80.6% in a historic cohort [22]. In a prospective study conducted in India higher rates of exclusive breastfeeding upon discharge in newborns of mothers without a COVID-19 infection (96.7%) were observed versus those of mothers who had an infection (31.6%) [23]. In contrast, in a study that included the participation of 17 countries, it was observed that at the time of discharge, 72.4% of mothers fed using exclusive breastfeeding, with the rates tending to decrease over the course of the study, although they did not find differences based on the mother having a COVID-19 infection [20].

The ten-step strategy carried out in BFHI-accredited hospitals has proven to be an effective measure to increase the rates of exclusive breastfeeding upon discharge [2, 24]. To date, in these types of hospitals, there is not enough evidence about the impact that a maternal COVID-19 infection at the time of the birth could have, even more so when the application of measures such as immediate skin-to-skin care or rooming-in have been reinstated after the initial controversy [5, 6]. In a study conducted in the first weeks of the pandemic in Spain [14], the rates of exclusive breastfeeding upon discharge for the newborns of mothers with a COVID-19 infection were 49.1% in BFHI-accredited hospitals versus 35.3% in non-accredited hospitals; figures that are lower than those obtained in our study due, primarily, to the initial difficulty found in adapting the perinatal care measures recommended by the WHO [7]. Likewise, in three BFHI-accredited hospitals in the first weeks of the pandemic, Popofsky et al. observed a reduction in the rates of exclusive breastfeeding in those mothers who had a COVID-19 infection [25]. Finally, in a retrospective study conducted in a BFHI-accredited maternity ward in the first three waves of the pandemic, a significant difference was observed in the rates of exclusive breastfeeding upon discharge in mothers with a COVID-19 infection at the time of the birth (70.7%) versus 86.2% in a control group [26].

In our study, the fact that we chose pandemic dates subsequent to those published in the literature is due to us wanting to assess the true impact a COVID-19 infection could have on exclusive breastfeeding upon discharge, while also taking into account that the hospitals included in this study routinely applied the steps established in the BFHI, and that the healthcare staff had high rates of vaccination, which made it less likely for the assessment of breastfeeding during the stay to be influenced by this aspect. We do not believe that a maternal COVID-19 infection is the cause of the differences observed per se as, at least up to now; it lacks biological plausibility, even more so when findings observed throughout the follow-up are not upheld, and as it can also be observed that the mothers have a sense of security when providing breastfeeding while applying the recommended hygiene measures. It is possible that although the mothers did not report differences in the various perinatal variables taken into account, the counseling activities conducted for supporting maternal breastfeeding were reduced (both in terms of duration and in the number of visits conducted during the hospital stay) in the group of mothers with a COVID-19 infection. Likewise, it is possible that other aspects that we have not taken into account in this study may have an impact, such as the presence of psycho-emotional distress. The presence of maternal psychological disorders, such as post-partum depression or anxiety have proven their impact in the reduction of breastfeeding rates [27, 28]. The pandemic situation or a COVID-19 infection itself entail a greater risk of presenting these types of disorders [16], and therefore, may have some type of influence on the findings observed. Nevertheless, and given the elevated risk of mothers with a COVID-19 infection diagnosed at the time of the birth not providing exclusive breastfeeding upon discharge to newborns (the risk being up to five times greater), it may be worth conducting further studies that take into account all the possible risk factors that could have an influence on the rates of exclusive breastfeeding upon discharge in these mother-infant pairs in order to assess the true impact that a COVID-19 infection at the time of the birth could have on breastfeeding.

In our study we also observed the elevated risk (up to 36 times greater) of not providing exclusive breastfeeding at the time of discharge in the case of mothers who had not previously practiced breastfeeding. It is of great relevance to provide adequate prenatal information and offer the necessary support with regard to initiating and maintaining satisfactory breastfeeding, as this can be a favoring factor toward mothers showing a higher tendency to provide breastfeeding in subsequent pregnancies [29] with the benefits that this has for the mother-baby pair [30–35]. In our study, there was a small percentage of mothers who used the support group resource, which, during the pandemic, modified their usual practices to include virtual care. This support tool has proven to be effective in providing support to mothers providing breastfeeding and who may have some type of difficulty. However, the support groups were also affected by the epidemiological situation, and in many cases had to stop their activities, which has resulted in a reduction in the number of resources available to support breastfeeding and which could have possible repercussions on the rates of breastfeeding in both the short- and long-term [36, 37]. Nevertheless, the use that some breastfeeding support groups made of mobile applications and social networks may be a useful tool to bear in mind for potential future pandemics. It is true that in our study we observed a greater follow-up of the newborns of mothers who had been diagnosed with a COVID-19 infection during pregnancy in the hospital setting, although this was due to the fact that, following the recommendations in force at that time in our neonatal scientific society [38], follow-up (on-site or by telephone) of these newborns was recommended, despite no short-term differences being observed in the rates of exclusive breastfeeding in this group of patients.

Other factors observed during our research which resulted in an increased risk of not providing exclusive breastfeeding upon discharge were birth via Cesarean and the birth weight. Evidence can be found in the literature about how birth via Cesarean results in a reduction in the rate of exclusive breastfeeding due, among other factors, to the reduced mobility of the mother, post-surgical pain, the different physiological context (above all in scheduled Cesareans) and the post-natal practices conducted (absence of immediate skin-to-skin contact) [39, 40]. During the period in which the study was being conducted, the indications for Cesarean were the routine indications and they were not affected by the maternal diagnosis of a COVID-19 infection. Despite this, the percentage of births that were completed via Cesarean was higher than the objectives established by the WHO [41] and therefore, taking into account the risk that this type of birth has with regard to reducing the rates of exclusive breastfeeding upon discharge (up to five times higher), the appropriate indication for Cesareans should be assessed. In the same manner, the influence that the weight of the newborn may have on breastfeeding rates has been described in various studies [42, 43]. In our research, although this was also observed, we believe it lacks clinical relevance.

We do not have sufficient statistical power to confirm that there are no differences in the rates of exclusive breastfeeding at three or six months of follow-up in either group, despite — at least in the first telephone contact — there being a greater proportion of mothers who had had a COVID-19 infection at the time of the birth, had gone back to work, and who reported a lower application of conditions aimed at facilitating the continuation of breastfeeding. Although exclusive breastfeeding rates are higher than those described at three and six months in the other studies conducted in our field [44–46], they are still not as high as the value determined by the WHO as one of their objectives for 2025 (exclusive breastfeeding rates of at least 50% at 6 months) [47]. With regard to the impact that the pandemic has had on the rates of exclusive breastfeeding at three and six months, we noted how in the study conducted by Maria *et* al on the newborns of mothers with a COVID-19 infection at the time of birth between March and June 2020, they obtained rates of exclusive breastfeeding at 3 months of 16.0% vs. 93.5% in the newborns of those mothers without a COVID-19 infection; differences that were influenced by the initial recommendation to separate the mother-infant pair and by the fear of transmitting the infection via breast milk [23]. In another study conducted in Thailand during the second wave of the COVID-19 pandemic, rates of exclusive breastfeeding at six months of 37.4% were obtained [48], figures that are similar to those obtained in our study and those found by Kwan et al. [49]. Although in our study the diagnosis of a COVID-19 infection did not have an impact in the mid- to long-term on the rates of exclusive breastfeeding, it is possible that other factors that we have not taken into account, since they were not the primary objective of the research, have had an influence in the reduction of exclusive breastfeeding rates observed over time, such as maternal obesity, smoking, and the use of assisted reproduction techniques, among others.

Within the limitations of the study, we found that the mother-infant pairs selected as the comparison group may not be representative of the mothers without a COVID-19 infection during pregnancy during this same period. Selection bias cannot be ruled out, given that the sample in the comparison group is a result of convenience sampling; despite having sufficient statistical power to expose potential differences Another limitation is the lower number of non-COVID mother-infant pairs prior to the COVID case due to the impossibility of obtaining the informed consent since they had been discharged before we were able to collect it. This is a study that has been conducted in a very select group of hospitals, all of which are BFHI-accredited, which although this limits its external validity, it does make it possible to avoid the influence that certain perinatal practices may have on exclusive breastfeeding rates. The fact of presenting with a COVID-19 infection during the first trimesters of pregnancy or during follow-up (either in the mother or the infant) was not taken into account, which could also have an influence on the results observed. The strengths of the study include its prospective nature, the temporary coexistence of both groups, and the fairly low number patients who were lost to follow-up.

## Conclusions

Mothers with a mild or asymptomatic COVID-19 infection at the time of the delivery had a lower probability of providing exclusive breastfeeding at the time of discharge from hospital in a group of BFHI-accredited hospitals. No previous practice of breastfeeding, having a Cesarean, and low birth weight were other risk factors observed in this study associated with not providing exclusive breastfeeding upon discharge.

Further research is required which, taking into account all the risk factors related to starting and maintaining exclusive breastfeeding, explores the true influence that a COVID-19 infection per se could have on the rates of exclusive breastfeeding in different periods.

## Data Availability

There are no identifying data in the manuscript. Data may be available from the principal investigator, Dr. Marín Gabriel, upon reasonable request.

## Abbreviations

AOR: Adjusted odds ratio
BF: Breastfeeding
BFHI: Baby Friendly Hospital Initiative
CI: Confidence interval
EBF: Exclusive breastfeeding
FF: Formula feeding
IHAN: Initiative for Humanizing Birth and Breastfeeding Care
IQR: Interquartile range
PCR: Polymerase chain reaction
UNICEF: United Nations Children’s Fund
WHO: World Health Organization
